# Bioinformatics and systems biology approaches to identify the effects of COVID-19 on neurodegenerative diseases: A review

**DOI:** 10.1097/MD.0000000000032100

**Published:** 2022-12-09

**Authors:** Fan Bu, Ruiqian Guan, Wanyu Wang, Zhao Liu, Shijie Yin, Yonghou Zhao, Jianbo Chai

**Affiliations:** a Heilongjiang University of Chinese Medicine, Haerbin, Heilongjiang Province, China; b Heilongjiang University of Chinese Medicine Affiliated Second Hospital, Haerbin, Heilongjiang Province, China.

**Keywords:** brain tissue, COVID-19, differentially expressed genes, immune response, neurodegenerative diseases

## Abstract

Severe acute respiratory syndrome coronavirus 2 (SARS-CoV-2), causing coronavirus disease (COVID-19), has been devastated by COVID-19 in an increasing number of countries and health care systems around the world since its announcement of a global pandemic on 11 March 2020. During the pandemic, emerging novel viral mutant variants have caused multiple outbreaks of COVID-19 around the world and are prone to genetic evolution, causing serious damage to human health. As confirmed cases of COVID-19 spread rapidly, there is evidence that SARS-CoV-2 infection involves the central nervous system (CNS) and peripheral nervous system (PNS), directly or indirectly damaging neurons and further leading to neurodegenerative diseases (ND), but the molecular mechanisms of ND and CVOID-19 are unknown. We employed transcriptomic profiling to detect several major diseases of ND: Alzheimer 's disease (AD), Parkinson' s disease (PD), and multiple sclerosis (MS) common pathways and molecular biomarkers in association with COVID-19, helping to understand the link between ND and COVID-19. There were 14, 30 and 19 differentially expressed genes (DEGs) between COVID-19 and Alzheimer 's disease (AD), Parkinson' s disease (PD) and multiple sclerosis (MS), respectively; enrichment analysis showed that MAPK, IL-17, PI3K-Akt and other signaling pathways were significantly expressed; the hub genes (HGs) of DEGs between ND and COVID-19 were CRH, SST, TAC1, SLC32A1, GAD2, GAD1, VIP and SYP. Analysis of transcriptome data suggests multiple co-morbid mechanisms between COVID-19 and AD, PD, and MS, providing new ideas and therapeutic strategies for clinical prevention and treatment of COVID-19 and ND.

**Key points**

• The AD, PD and MS had some significant common genes compared with the COVID-19 to assess the distinct genetic mechanism involved.
• Gene set enrichment–based analysis predicts Gene ontology terms for among AD, PD, MS and COVID-19 affected lung cells, and hub gene identification makes the prediction of drug compounds even more useful.
• Protein–protein interactions network-based analysis helps determine the definite genes related to AD, PD, MS, and COVID 19. It can lead us to their coexpression partners about normal and disease states and assess risk factors.
• Transcription factors–genes interaction and DEGs–miRNAs coregulatory network with common DEGs also identified on the datasets to find the transcriptional and posttranscriptional regulators of the common DEGs.
• The protein–drug interactions suggested 10 potential chemical compounds against COVID-19.

## 1. Introduction

Severe Acute Respiratory Syndrome Coronavirus 2 (SARS-CoV-2) is a respiratory disease, thus causing a novel coronavirus disease (COVID-19) pandemic in 2019. As of April 20, 2022, world health organization received data showing that there are more than 504.4 million confirmed cases of COVID-19 and more than 6.2 million COVID-19-related deaths worldwide.^[1]^ Patients presenting with neurological symptoms during the acute phase of COVID-19 infection are not an exception but have a significant trend. SARS-CoV-2 is a highly neuroinvasive neurotropic virus that invades cells via an angiotensin-converting enzyme 2 (ACE2) receptor-driven pathway.^[2]^ SARS-CoV-2-mediated neuroinvasion, neuroinflammation and blood-brain barrier (BBB) dysfunction may contribute to the development of neurodegenerative diseases (ND). There is growing evidence of an interaction between the pathology of central nervous system (CNS) disease and COVID-19,^[3]^ with the potential to induce permanent sequelae of CNS through different mechanisms.^[4]^ Twenty-five percent of patients develop neurological lesions due to direct COVID-19 invasion of the CNS,^[5]^ which further leads to the development of ND.^[6]^ As to whether SARS-CoV-2 virus mimics the coronavirus to invade peripheral nerve endings as a springboard and subsequently enter the CNS via the trans-synaptic pathway, this conjecture remains to be confirmed.^[7]^ ND is the most common age-related chronic neurological disease,^[8]^ and global aging and increasing human lifespan pose a non-negligible problem – the increasing prevalence of ND, which kills nearly 3 million people worldwide each year.^[9]^ Within the age range of the COVID-19 infected population, older patients are most severely affected,^[10]^ and thus the age range of ND patients is highly significant in the area of overlap with the age at risk for COVID-19 infection. There is now evidence that patients with COVID-19 may induce the development of ND and exacerbate associated symptoms.^[11]^ ND consists mainly of Alzheimer’s disease (AD) and Parkinsonism (PD),^[12]^ in addition to multiple sclerosis (MS), amyotrophic lateral sclerosis,^[13]^ frontotemporal dementia, and Huntington’s chorea.^[14]^

Several studies have investigated the possible association between COVID-19 and various types of diseases in ND. AD typically leads to chronic brain inflammation and BBB damage^[15]^ and is more susceptible to penetration by viruses with high neuroinvasive potential such as SARS-CoV-2. However, the degree of BBB damage caused by AD needs further study. Model predictions suggest that the presence or absence of AD is strongly associated with SARS-CoV-2 infectivity. Compared to non-AD patients, AD patients are not only more susceptible to SARS-CoV-2 infection, but also have a higher risk of death following infection.^[16,17]^ We observed that Yu Y’s patient health records were all derived from U.K. and may not be representative of all humans. In the same model, PD was also found to be associated with an increased risk of SARS-CoV-2 infectivity, but not with mortality, a finding that is consistent with the results of another study.^[18]^ Yet, other studies have shown that patients with PD, while highly susceptible to COVID-19 leading to worsening symptoms, are additionally likely to have increased mortality following infection, especially in the late stages of the disease.^[19]^ A large community-based study found that 57% of MS patients experienced worsening of MS during COVID-19 infection and 20% developed new MS symptoms, suggesting that COVID-19 infection is associated with the exacerbation of MS.^[20]^ Although the neurological outcome of patients with MS and related diseases (MSRD) after COVID-19 is unknown, it can be determined that the severity of COVID-19 is associated with new or worsening neurological symptoms in patients with MS and MSRD.^[21]^

To understand how COVID-19 affects these ND patients and to identify potentially effective drugs for ND patients suffering from COVID-19, thereby reducing the risk of hospitalization or death, datasets of COVID-19 and 3 major ND were selected to obtain differentially expressed genes (DEGs). With the help of these DEGs, bioinformatics and systems biology approaches were used to analyze the underlying molecular mechanisms and identify some potentially effective drugs with relevant targets for the treatment of COVID-19 and ND.

## 2. Materials and Methods

### 2.1. Data acquisition

Common morbidity complexity and common genetic correlations between COVID-19 and ND were investigated using bioinformatics as well as systems biology approaches from microarray and RNA-Seq datasets from the National Center for Biotechnology Information (NCBI) database GEO (https://www.ncbi.nlm.nih.gov/geo). Although classical experimental methods are always used, tissue sources are usually not uniform.^[22,23]^ We improved this in our study.

Raw human gene expression datasets for COVID-19, AD, PD and MS were collected. The GEO accession number for the COVID-19 dataset is GSE188847. The accession numbers for the ND dataset are GSE104704 and GSE159699 (AD patients and healthy controls), GSE68719 and GSE135036 (PD patients and healthy controls) and GSE123496 and GSE138614 (MS patients and healthy controls). See Table 1.

**Table 1:** COVID-19 and ND datasets.

Disease	ID	Platforms	Organization	Control group	Case group
COVID-19	GSE188847	GPL24676	Essence	12	12
AD	GSE104704	GPL18573	Essence	18	12
AD	GSE159699	GPL18573	Essence	18	12
PD	GSE68719	GPL11154	Essence	44	29
PD	GSE135036	GPL18573	Essence	12	24
MS	GSE123496	GPL21290	essence	25	25
MS	GSE138614	GPL21697	Essence	49	20

AD = Alzheimer’s disease, COVID-19 = coronavirus disease, MS= multiple sclerosis, ND = neurodegenerative diseases, PD= Parkinson’s disease.

### 2.2. Identification of DEGs

To identify DEGs in the dataset, after converting some counts data into fpkm data via SangerBox^[24–26]^ (http://vip.sangerbox.com/), a difference-in-difference analysis using limma R and combined with the Benjamini-Hochberg false discovery rate method was used to screen for statistically significant genes and limit false positives.^[27,28]^ Genes with a screening-adjusted *P* ≤ .05 and log_2_ FC ≥ 0.585 (fold change = 1.5)^[29–31]^ were identified as DEGs. Genes common to both datasets of the same tissue for the same disease were selected for ND as DEGs for that disease to improve precision.^[32]^ DEGs were obtained for the COVID-19 dataset versus the ND dataset using the jvenn analysis tool (http://jvenn.toulouse.inra.fr/app/example.html). Common DEGs in COVID-19 immune and ND were determined by ImmPort (http://www.immport.org/).

### 2.3. Gene ontology (GO) and kyoto encyclopedia of genes and genomes (KEGG) analysis

GO and KEGG analyses of common DEGs were performed using SangerBox,^[26,33]^ and the top 5 important pathways were plotted in GO analysis based on *P*-value for biological process, molecular function and cellular component, respectively; the top 10 important pathways were plotted in KEGG of the top 5 important pathways in GO analysis; the top 10 important pathways were plotted in KEGG.

### 2.4. Protein-protein interaction (PPI) network analysis

To explore associations between diseases with the help of PPI,^[34]^ DEGs were uploaded to STRING (www.string-db.org) and a PPI network of common DEGs was constructed by setting a medium confidence score (0.4), hiding unassociated protein nodes. Cytoscape was used to further visualize the degree of lineage expression binding between gene nodes.

### 2.5. Hub gene (HG) extraction and submodule analysis

The top 8 (25%) common DEGs were identified as HGs from the network of common DEGs using Cytoscape plugin Cytohubba, with maximal clique centrality (MCC) as the criterion,^[35–37]^ and their ranks are presented as color gradients in the graph. The shortest reachable paths between central genes were ranked using Cytohubba’s neighbor-neighbor ranking feature.

### 2.6. Gene regulatory network (GRN) analysis

The NetworAnalyst platform (https://www.networkanalyst.ca/)^[38]^ was used for meta-analysis of gene expression data and in-depth exploration of biological mechanisms and roles. The JASPAR database^[39]^ was selected to build a transcription factors (TFs)-common DEGs common expression network; the Tarbase database^[40]^ was selected to build a minimal common expression network of mRNA-common DEGs, and micro RNAs (miRNAs) interacting with common DEGs can be used to track miRNAs that interact with gene transcripts binding to affect protein expression; the DisGeNET^[41]^ database was selected to establish a disease-HG common expression network to predict HG-related diseases and complications.

### 2.7. Treatment drug projections

Assessment of protein-drug interactions is important to understand the structural features recommended for receptor sensitivity.^[42]^ Drug molecules were predicted by the Enrichr platform (https://maayanlab.cloud/Enrichr/) in the DSigDB database^[43]^ based on the common DEGs of COVID-19 and ND, and the top 5 compounds were extracted by root binding degree.

## 3. Results

### 3.1. Common DEGs in COVID-19 and ND

The interrelationship and significance of COVID-19 with ND was investigated by analyzing the human RNA-Seq dataset and microarray dataset from NCBI. 420 DEGs (320 up-regulated genes, 100 down-regulated genes) were extracted in the COVID-19 dataset, 260 DEGs in the 2 AD datasets; 71 DEGs in the 2 PD datasets; and 32 DEGs in the 2 MS datasets. See Table 2.

**Table 2:** DEGs of COVID-19 and ND.

Disease	ID	Up	Down	common diff
COVID-19	GSE188847	100	320	420
AD	GSE104704	573	261	260
AD	GSE159699	295	40	
PD	GSE68719	1415	851	71
PD	GSE135036	44	128	
MS	GSE123496	530	32	32
MS	GSE138614	889	861	

AD = Alzheimer’s disease, COVID-19 = coronavirus disease, DEG = differentially expressed gene, MS= multiple sclerosis, ND = neurodegenerative diseases, PD= Parkinson’s disease.

### 3.2. Common DEGs for COVID-19, AD, PD, and MS immune responses

**Figure 1.**
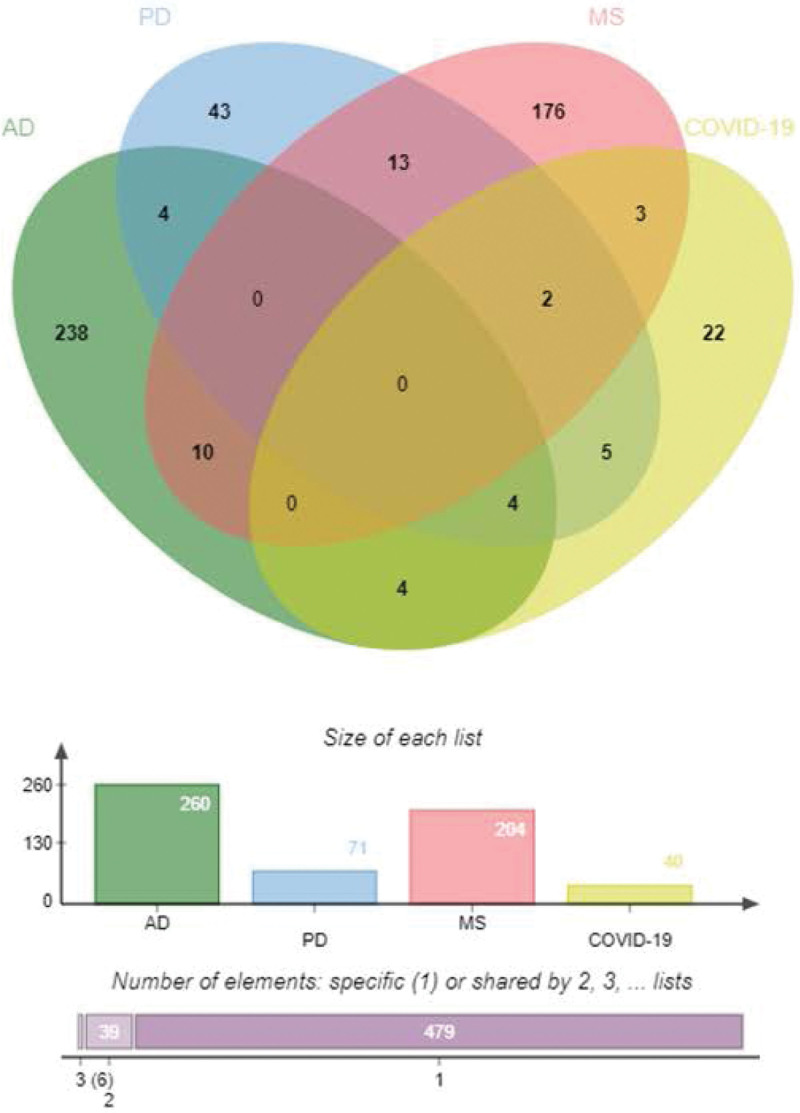
Common DEGs in COVID-19 and ND. COVID-19 = coronavirus disease, DEG = differentially expressed gene, ND = neurodegenerative diseases.

**Figure 2.**
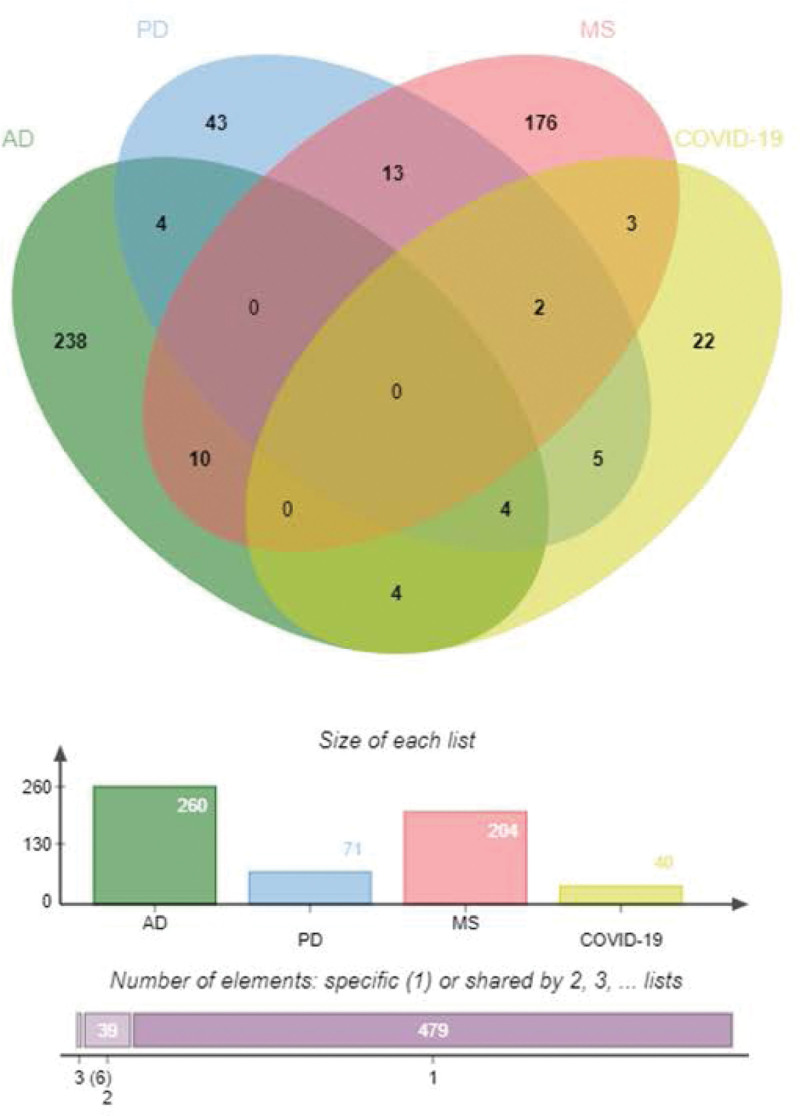
Immunological Common DEGs of COVID-19 and ND. COVID-19 = coronavirus disease, DEG = differentially expressed gene, ND = neurodegenerative diseases.

We identified common DEGs from the immune response genomes of COVID-19, AD, PD, and MS patients (Fig. 1, Table 3), plotted heat maps showing the relationship between these common DEGs based on sample expression and found that a significant proportion of the common DEGs belonged to the immune common DEGs of COVID-19 with ND (Fig. 2, Table 4).

**Table 3:** Common DEGs of COVID-19 and ND.

Disease	Common DEGs
COVID-19 & AD	14
COVID-19 & PD	30
COVID-19 & MS	19

AD = Alzheimer’s disease, COVID-19 = coronavirus disease, DEG = differentially expressed gene, MS= multiple sclerosis, ND = neurodegenerative diseases, PD= Parkinson’s disease.

**Table 4:** Immunological common DEGs of COVID-19 and ND.

Disease	Number	Common DEGs
COVID-19 & AD	8	TAC1, SST, IL17RB, CRH, PCSK1, SCG2, VGF, NFKBIA
COVID-19 & PD	11	sst, s100a9, s100a11, vip, mchr2, crh, pcsk1, pak1, osmr, vgf, tlr2
COVID-19 & MS	5	s100a11, plscr1, ifitm1, tlr2, s100a10

AD = Alzheimer’s disease, COVID-19 = coronavirus disease, DEG = differentially expressed gene, MS= multiple sclerosis, ND = neurodegenerative diseases, PD= Parkinson’s disease.

### 3.3. GO and KEGG analysis

**Figure 3.**
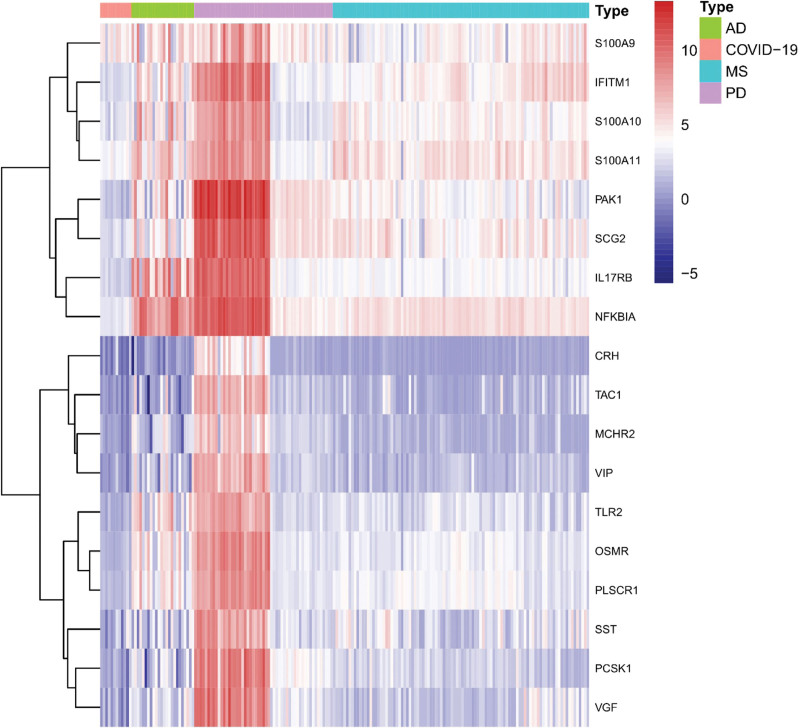
(A) KEGG of COVID-19 with AD common DEGs in brain tissue and immune samples, (B) KEGG of COVID-19 with PD common DEGs in brain tissue and immune samples, (C) KEGG of COVID-19 with MS common DEGs in brain tissue and immune samples, (D) GO of COVID-19 with AD common DEGs in brain tissue and immune samples, (E) GO of COVID-19 with PD common DEGs in brain tissue and immune samples, and (F) GO of COVID-19 with MS common DEGs in brain tissue and immune samples. AD = Alzheimer’s disease, COVID-19 = coronavirus disease, DEG = differentially expressed gene, GO = gene ontology, KEGG = Kyoto Encyclopedia of Genes and Genomes, MS= multiple sclerosis, ND = neurodegenerative diseases, PD= Parkinson’s disease.

The functional analysis of COVID-19 and ND common DEGs identified the related pathways of COVID-19 and AD, PD and MS common DEGs, and analyzed the possible roles of these genes in the related pathways. After integrating relevant data sources, the pathways were mapped as shown in Figure 3. Among the KEGGs, neuroactive ligand-receptor interactions, taurine and hypotaurine metabolism, and mineral uptake were the most significantly enriched pathways in AD, PD, and MS, respectively. And there were also mechanisms associated with the pathogenesis of lung infections, i.e., Legionellosis, which causes infections with pneumonia as the main cause,^[44]^ as shown in Figure 3(A–C). In pathways closely related to the inflammatory response, the cAMP signaling pathway, IL-17 signaling pathway, MAPK signaling pathway, and PI3K-Akt signaling pathway were closely associated with COVID-19 and ND. In the GO functional analysis, Figure 3(D–F) shows the top 5 important pathways expressed among biological process, molecular function, and cellular component, respectively, in the common DEGs of COVID-19 and ND in GO analysis. Protein secretion, peptide secretion, cellular secretion, and secretory vesicles are repeatedly expressed in COVID-19 and ND. Peptide hormone activity and peptide hormone receptor binding were significantly enriched in AD and PD. In addition, some of the pathways are associated with various viral pathogenesis, such as regulation of viral genome replication and replication, regulation of viral life cycle.

See Figure 3 for an enrichment analysis of COVID-19 with ND common DEGs in brain tissue and immune samples. (A-C) KEGG pathways of AD, PD and MS, respectively; (D-F) GO pathways of ND, respectively.

### 3.4. PPI network analysis and HG screening

**Figure 4.**
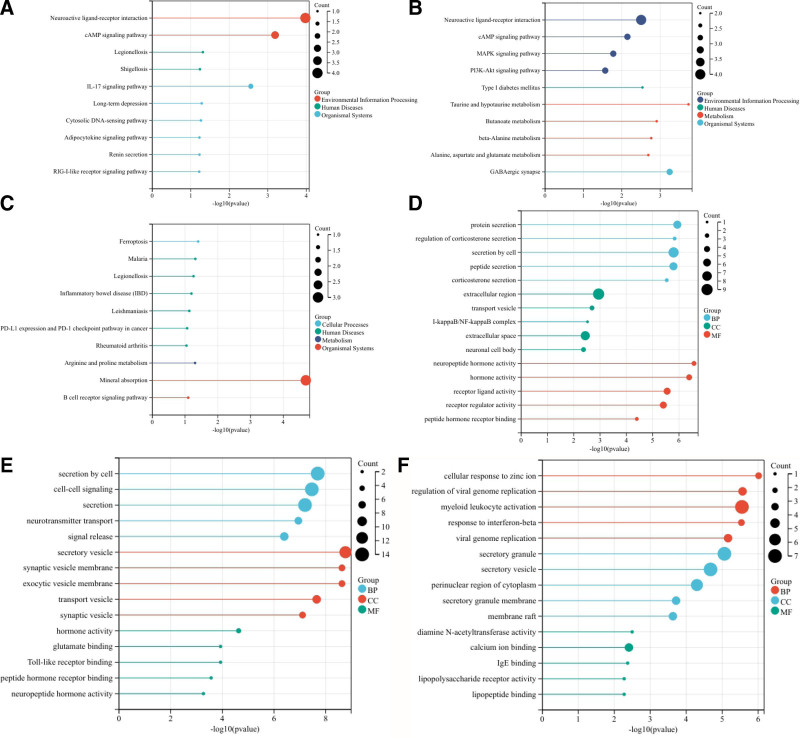
(A) PPI network of COVID-19 and ND common DEGs and (B) HG network of COVID-19 and ND common DEGs. COVID-19 = coronavirus disease, DEG = differentially expressed gene, HG = hub gene, ND = neurodegenerative diseases, PPI = protein-protein interaction network.

Create a PPI network showing the common DEGs between COVID-19 and AP, PD and MS, see Figure 4A the common DEGs. The PPI network consists of 40 nodes and 70 edges. The common DEGs with interconnections were analyzed using Cytohubba to filter the top 8 (25%) DEGs as HGs by MCC: CRH, SST, TAC1, SLC32A1, GAD2, GAD1, VIP, and SYP. These 8 HGs have the highest connectivity in the PPI network and any 2 have interconnections. See Figure 4B.

### 3.5. GRN analysis

**Figure 5.**
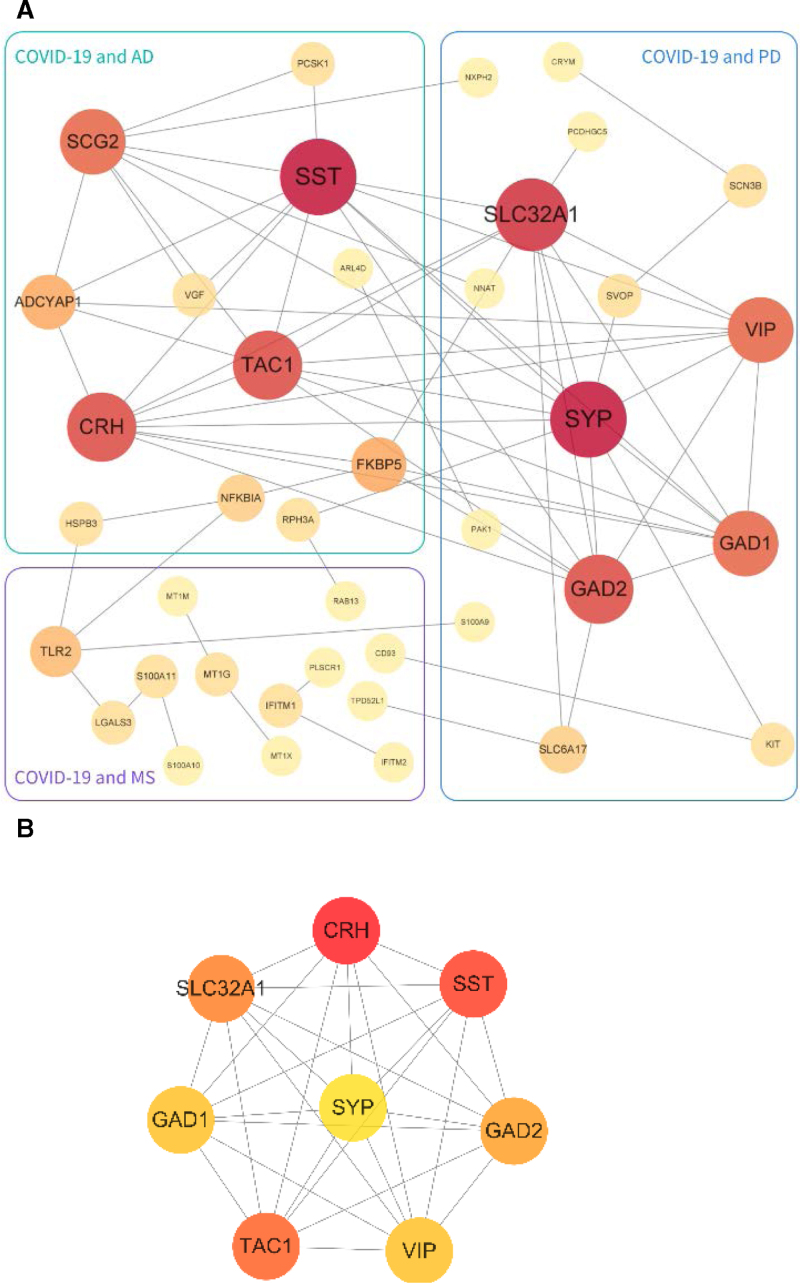
(A) DEGs-miRNAs network for COVID-19 and AD, (B) DEGs-miRNAs network for COVID-19 and PD, (C) DEGs-miRNAs network for COVID-19 and MS, (D) DEGs-TFs network for COVID-19 and AD, (E) DEGs-TFs network for COVID-19 and PD, and (F) DEGs-TFs network for COVID-19 and MS. AD = Alzheimer’s disease, COVID-19 = coronavirus disease, DEG = differentially expressed gene, MS= multiple sclerosis, miRNA = micro RNA, PD= Parkinson’s disease, TFS = transcription factors.

The association between common DEGs and miRNAs, DEGs and TFs was shown by analyzing their interaction networks on the NetworkAnalyst platform. Figure 5(A–C) shows the DEGs-miRNAs network of COVID-19 and AD, PD and MS, respectively. Figure 5(D–F) shows the DEGs-TFs network between COVID-19 and ND. Circles indicate DEGs, diamonds indicate TFs, and squares indicate miRNAs. Graph size and color depth indicate node criticality.

In the DEGs-miRNAs interaction network of COVID-19 and AD, FKBP5, SEZ6L2, VGF, PCSK1 and hsa-miR-1-3p, hsa-miR-27a-3p, hsa-miR-155-5p, hsa-miR-34a-5p were significantly expressed, see Figure 5A. In the DEGs-miRNAs interaction network of COVID -19 and the DEGs-TFs interaction network of PD, STOM, IFITM2, SAT1 and PLP2 were significantly expressed, and FOXC1, GATA2 and RELA were important partial TFs. See Figure 5E.

### 3.6. Drug forecasting

The transcriptional features in the DSigDB database predicted drug molecules based on the common DEGs of COVID-19 with ND and showed the top 5 compounds based on the binding degree.

### 3.7. Identification of disease associations

**Figure 6.**
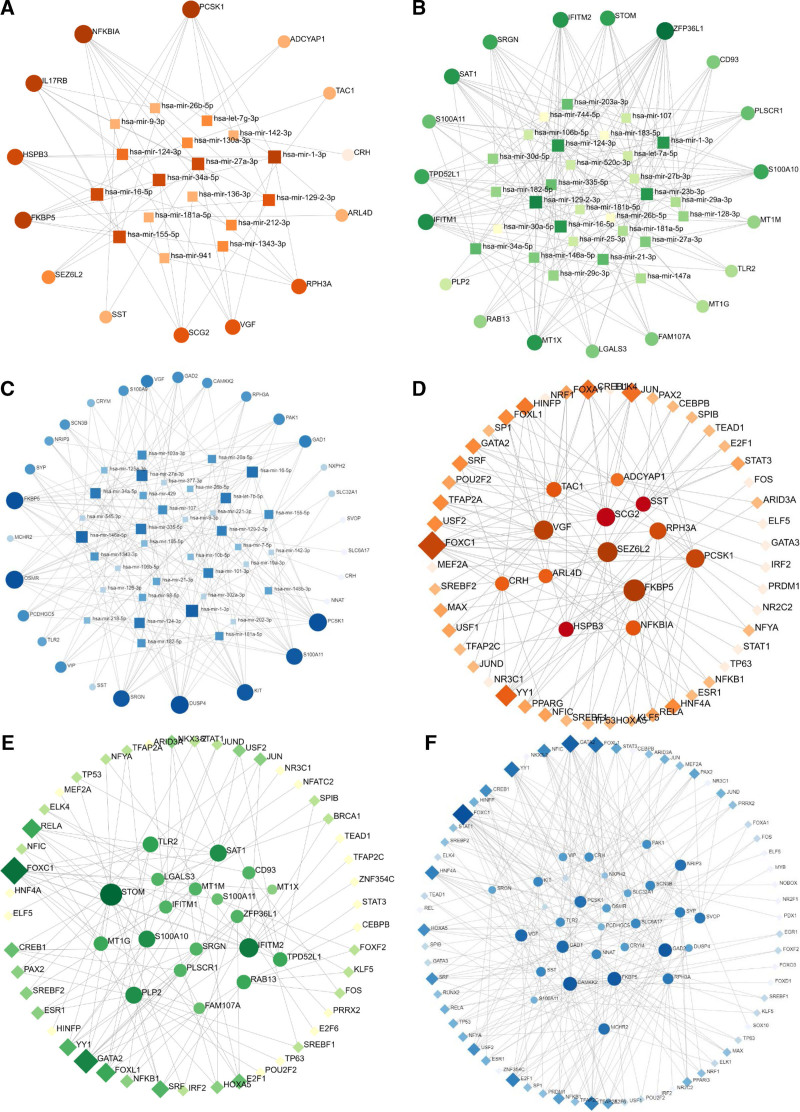
COVID-19 and ND for common DEGs disease prediction. COVID-19 = coronavirus disease, DEG = differentially expressed gene, ND = neurodegenerative diseases.

From NetworkAnalyst’s analysis of genetic disease associations, neuropsychiatric disorders such as major depressive disorder, schizophrenia, mood disorders, and bipolar disorder were found to be closely related to the hub gene, and the associations between genetic disorders are shown in Figure 6.

## 4. Discussion

Transcriptomic data can better uncover studies related to COVID-19 comorbidity.^[45,46]^ Barh D’s study has been a great help in exploring the association of COVID-19 with other diseases, but unfortunately, experimental validation is lacking. The core of this paper is to explore the pathogenesis and genetic mechanisms between COVID-19 and ND from different biological perspectives. Global aging and increasing human lifespan have brought about an inescapable problem—the increasing prevalence of ND, which kills nearly 3 million people worldwide each year.^[9]^ Fu^[3]^ described several potential pathways for COVID-19 to invade the brain.

Olfactory and gustatory degeneration is one of the main symptoms of COVID-19, and although the prevalence of chemosensory dysfunction in COVID-19 is expressed with significant differences between populations and races, with the prevalence of chemosensory dysfunction in Western populations being 3 times higher than in Eastern regions, in case reports of COVID-19 patients worldwide, olfactory loss, gustatory loss and any chemosensory loss all exceeded 40% of the total number of patients,^[47]^ with pendant cells in the olfactory epithelium playing a key role. In a previous study, it was shown that SARS-CoV virus was able to induce neuronal cell death in mice by invading the brain through the nasal and olfactory epithelia.^[48]^ As for SARS-CoV-2 virus, it is able to readily enter the pendent cells and cause massive degeneration of the olfactory epithelium and extensive loss of olfactory cilia,^[49]^ while there is also indirect evidence that SARS-CoV-2 virus can transcend the pendent cells and enter the brain retrogradely through the neurons of the olfactory system.^[50]^ In conjunction with experiments in mice, the possibility exists that the virus is transferred from the olfactory epithelium to the brain via the cribriform plate.^[51]^ Although satisfactory results have been obtained, more human-related experiments are still needed to ensure the reliability of the results. Although Meinhardt et al^[52]^ suggested that the neuro-mucosal interface could be a potential pathway for SARS-CoV-2 neural invasion, the nasal-brain pathway has not been widely recognized. What has been able to be established, however, is that SARS-CoV-2 is able to reach the brain through the nasal cavity and affect the normal physiological activity of the CNS,^[53]^ and an autopsy report from another study was able to provide strong support for this theory.^[54]^ Unfortunately, it cannot be supported by autopsy sample data from a large number of patients.

Brain endothelial cells (BEC), the major unit of the BBB, help regulate substrate migration into and out of the CNS and are a major target of SARS-CoV-2. Several reports have confirmed that SARS-CoV-2 viruses are capable of invading endothelial cells and neurons,^[55,56]^ leading to BEC and BBB damage and apoptosis,^[53,57]^ and that BBB dysfunction occurring in response to SARS-CoV-2 infection may depend on interactions with the immune system, as well as with the virus and its components. But some of the results are unsatisfactory, and more in-depth studies on neurons are needed. SARS- CoV-2 causes a systemic inflammatory response resulting in elevated plasma cytokine counts in the brain and blood,^[58]^ where chemokines such as IL-6, TNF-α and cytokines such as the acute phase protein CRP, depending on the severity of inflammation, can disrupt the BBB paracellular and transcytosis.^[59,60]^Another pathological manifestation caused by SARS-CoV-2 -hypoxia,^[61]^ induces BBB destruction, which is thought to be the mechanism by which viral and infected immune cells enter the CNS of the brain.^[62]^ Animal models can indeed more easily illustrate how SARS-CoV-2 enters the brain, but data generated using transgenic ACE2 mice driven by artificial promoter systems should be interpreted when considering the translatability of the results to humans.

**Figure 7.**
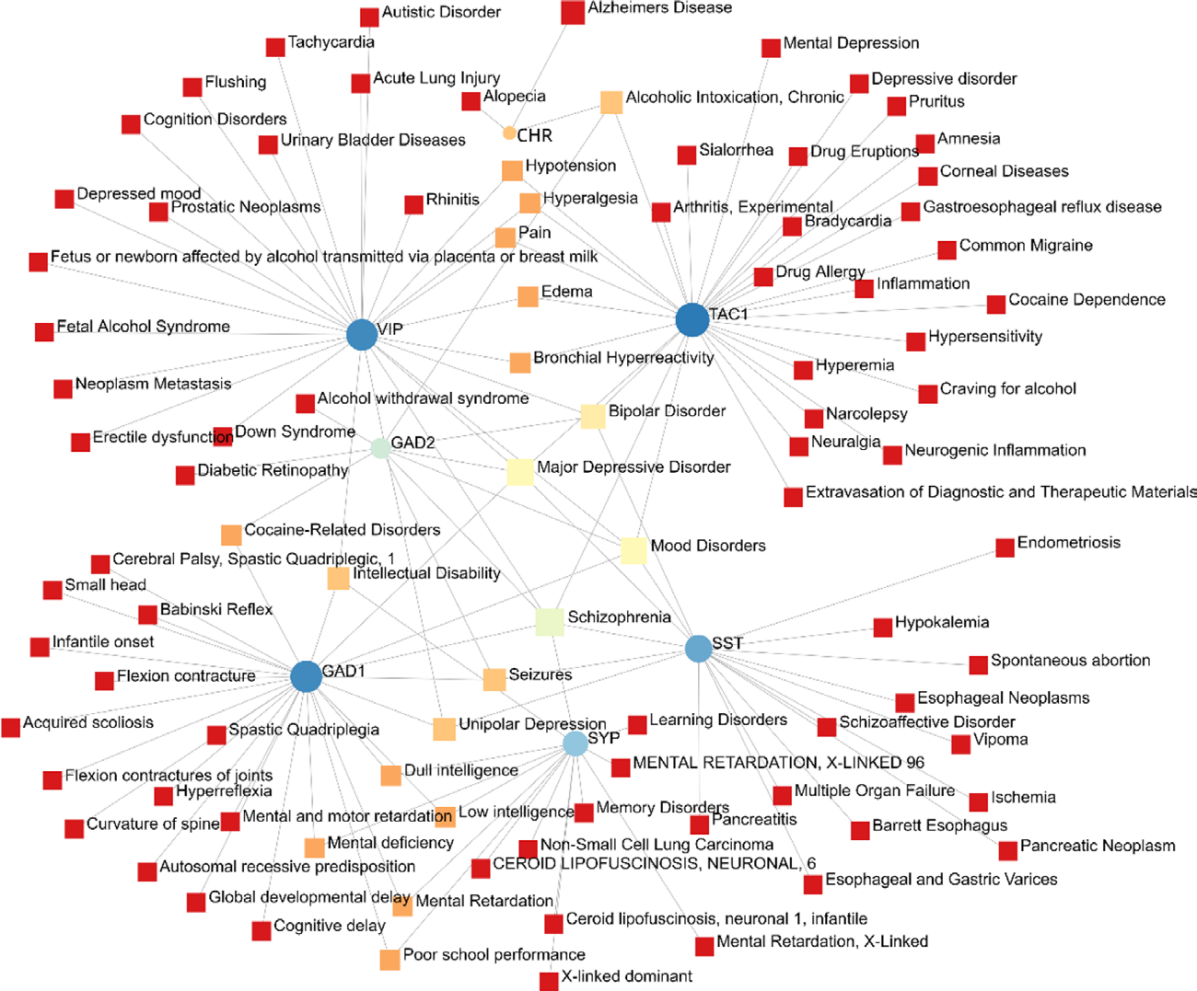
Expression of immune common DEGs of COVID-19 and ND. COVID-19 = coronavirus disease, DEG = differentially expressed gene, ND = neurodegenerative diseases.

A comprehensive analysis of multiple transcriptome data revealed 14, 30, and 19 common DEGs between COVID-19 and AD, PD, and MS, respectively, in the brain tissue transcriptome dataset, and nearly half of the DEGs for ND were found to be involved in immunology in the intersection of immune DEGs with COVID-19 identified by ImmPort. Of these, there were 4 common DEGs for COVID-19 immunogene-AD-PD (SST, CRH, PCSK1, VGF) and 2 common DEGs for COVID-19 immune-PD-MS (S100A11 and TLR2) (Fig. 7).

In the present study TLR2 was an upregulated factor in the association dataset, and there is growing evidence to support a possible role of TLR2 as a potential SARS-CoV-2 receptor in the CNS in the pathogenesis of SARS-CoV-2.

The surface protein of SARS-CoV-2 is able to express PAMP and induce upregulation of inflammatory factors via TLR2 and TLR4 in rodent models.^[63]^ Prophylactic administration of TLR2 receptor agonists was able to activate innate immune cells and lung epithelial cells, inhibit excessive inflammation and tissue damage, as well as maintain the integrity of local epithelial barrier function.^[64]^ Proud et al^[65]^ similarly found in a SARS-CoV-2 ferret infection model that prophylactic administration of the TLR2/6 agonist INNA-051 via intranasal reduce viral RNA levels in the upper respiratory tract and control SARS-CoV-2 transmission. In addition, TLR2 senses the envelope (E) protein of SARS-CoV-2 and is thus induced to activate by the latter, leading to an inflammatory response,^[66]^ and downstream signaling molecules of TLR2 are significantly increased in patients with severe SARS-CoV-2 infection. TLR2 inhibitors also produce inhibition of the inflammatory response to SARS-CoV-2 infection in human leukocytes.

The role of TLR2 as an innate immune receptor in ND has long been established.^[67,68]^ Compared to healthy brain samples with very low TLR2 expression, autopsy analysis of brain samples from PD patients showed increased TLR2 expression in neurons and microglia in different brain regions,^[69]^ which is consistent with the results of blood samples tested from PD and healthy patients. But more systematic studies are needed on microglial response.^[70]^ In addition, TLR2 expression in specific brain regions was simultaneously positively correlated with disease stage.^[71]^ In mouse models of AD, as abnormal accumulation of amyloid β peptide (Aβ) and tau protein causes cognitive impairment in AD, cognitive function can be improved by inhibiting TLR2 function, which effectively downgrades microglia, astrocytes, Aβ plaque deposition and phosphorylated tau accumulation in brain regions.^[72]^ Hossai^[73]^ observed that, during infection activation of TLR1/2 exogenous dimers may exacerbate functional defects in regulatory T cells in MS, suggesting that TLR2 may be a target for the treatment of MS.

Excessive peripheral immune responses resulting from persistent SARS-CoV-2 infection can induce damage to the BBB,^[74]^ and more viruses can penetrate the CNS through the damaged barrier, thereby increasing the likelihood of viral binding to TLR2. Szabo^[75]^ speculated that SARS-CoV-2 in brain tissue, particularly the E protein, induces activation of TLR2 in microglia This increases TLR2 susceptibility to Aβ and induces neuroinflammation. tLR2 is also expressed in neurons, and long-term SARS-CoV-2 infection may induce neuronal TLR2 activation in the brain, and induction of neuronal TLR2 is closely associated with transmission and propagation between neurons in pathological alpha syndrome, which is associated with disease progression.^[76]^ Thus, E protein induction of neuronal TLR2 susceptibility by SARS-CoV-2 may lead to deposition of aberrant proteins in cells, thereby affecting COVID-19 episodes and accelerating ND progression.

Another group of upregulated genes, ND and COVID-19 common DEGs, S100A9, S100A10 and S100A11, belong to the same S100A family that regulates the inflammatory response^[77]^ and whose mediated signaling pathways are involved in the pathogenesis of ND and COVID-19.^[78,79]^ Zahra^[80]^ observed that, compared to healthy controls, mRNA expression of S100A9 and S100A10 was significantly upregulated in the peripheral blood of COVID-19 critically ill patients (FC > 1.8, *P* < .001).ROC curves calculated AUCs of 0.80 (95% CI 0.67–0.93, *P *= .002) and 0.71 (95% CI 0.56–0.85, *P *= .010) to determine the sensitivity and specificity of these molecules for COVID-19. Thus, S100A can serve not only as a potential clinical biomarker but also as a potential therapeutic target.^[81]^

GO analysis revealed pathways related to protein secretion, cellular secretion, and receptor ligand activity in AD and COVID-19. Toll-like receptors (TLRs) are significantly expressed in COVID-19 and PD, and their major members include TLR2, TLR3, TLR4, TLR6, TLR7, TLR8, and TLR9.TLR2 has been previously described. TLRs belongs to the innate immune receptors, mainly located in microglia, and is involved in the pathogenesis of PD as a promoter of the immune/neuroinflammatory response. Most studies have shown that TLR expression is increased in the brain and peripheral blood cells of PD samples, that TLR-mediated neuroinflammation may lead to dopaminergic neurological loss in PD patients, and that the TLR signaling pathway has been recognized as a possible cofactor in the pathogenesis of COVID-19.^[82,83]^ TLR4 activation, generated by SARS-CoV-2 S protein-TLR4 interaction, promotes ACE2 receptor expression in alveolar cells, thereby increasing infectivity and viral load, and TLR4 is currently receiving widespread attention as a therapeutic target for COVID-19 intervention.^[84]^ activation of TLR7/8 can cause a strong pro-inflammatory response during acute lung injury.^[64]^ In MS and COVID-19 enrichment analyses, the regulation of viral genome replication was also found to be highly enriched with the regulation of replication and viral life cycle, which could be a potential mechanism affecting the progression of disease in MS patients after SARS-CoV-2 infection.^[85]^ The analysis also identified multiple cellular signaling pathways between diseases, including cAMP, adipocytokines, IL-17, MAPK, PI3K-Akt, etc. The cAMP signaling pathway is involved in regulating cellular signaling and various physiological and pathological activities and has been applied in the treatment of neuropsychiatric disorders.^[86]^ cAMP-PKA pathway plays a role in neuronal growth and development, synaptic plasticity by regulating, neurogenesis and memory consolidation, plays a unique role in CNS and is critical for a large number of cellular functions including neuroplasticity and neuroprotection, and is a potential strategy for the treatment of ND. Dopamine D1 receptor agonists inhibit NLRP3 inflammatory vesicle activation by stimulating cAMP synthesis, and increased cAMP levels during inflammation inhibit the production of pro-inflammatory cytokines, stimulate the formation of anti-inflammatory factors and inhibit the development of inflammation.^[87]^IL-17 signaling pathways are involved in and play a key role in the pathogenesis of autoimmune diseases and ND^[88,89]^ and are also involved in carrying out multiple cancer modulation.^[90–93]^ Studies have shown that activation of the IL-17 signaling pathway is strongly associated with increased severity of viral respiratory infections and eventual inflammatory side effects,^[94]^ and that SARS-CoV-2 infection causes stronger activation of IL-17 signaling than other respiratory viruses.^[95]^ With a better understanding of the disease, the interaction of adipocytokine signaling pathways with COVID-19 has received widespread attention, and enhanced ACE2 expression induced by adipocytokine dysregulation, preexisting endothelial dysfunction and a procoagulant state in the metabolic syndrome may play a crucial role in the development of severe COVID-19.^[96]^ In addition, a number of potential pathways have been identified, such as neuroactive ligand-receptor interactions^[97–99]^ and renin secretion.^[100–102]^ However, we have identified some issues in the relevant literature. Han Z’s study included a non-randomized controlled trial, so the selected samples will inevitably have some subjectivity. Some studies lack direct experimental evidence, and therefore, this may be our future experimental direction.

The PPI network allows systematic analysis of protein interactions and understanding of the physiological mechanisms of disease states. In the present study, the 8 HGs screened were interrelated to an excellent degree. VIP, one of the HGs, showed a protective effect in SARS-CoV-2 infection. In patients with severe COVID-19, elevated plasma levels of the immunomodulatory neuropeptide VIP were associated with a reduction in inflammatory mediators and survival in these patients. In vitro, VIP inhibited SARS-CoV-2 RNA content in human monocytes and the production and replication of pro-inflammatory mediators and viruses in lung epithelial cells. This may be a consequence of the intrinsic regulation of inflammatory mediators and TFs directly and indirectly involved in viral replication by VIP.^[103]^ VIP prevented in monocytes the SARS-CoV-2-induced activation of NF-kB and SREBP1 and SREBP2, TFs involved in the pro-inflammatory response and lipid metabolism, respectively, and the activation of CREB (CREB is a transcription factor with anti-apoptotic activity and a transcription factor with NF-kB negative regulator).^[104]^ Specific inhibition of NF-kB and SREBP1/2 by VIP recapitulates anti-inflammatory, anti-viral and cell death protective effects.^[103,105,106]^ VIP exerts protective effects in cellular and animal models of different ND. In AD models, VIP prevents Aβ-induced neurodegeneration, inhibits the secretion of pro-inflammatory interleukins and neurotoxic drugs in microglia and induces Aβ phagocytosis.^[107]^ Alarmingly, the broad consensus that Aβ is a marker has been shocked recently, and relevant conclusions need to be debated. In PD neurons and animal models, VIP has been shown to exert an indirect protective effect by inhibiting microglia activation and astroglial cell proliferation in PD.^[108]^ In MS models, VIP exhibits not only potent anti-inflammatory effects but also significant neurotrophic and neuroprotective effects. Curiously, we did not see experiments in recent years.^[109]^ CRH, SST and TAC1 are all closely related to neurotransmission and secretion in brain tissue and are associated with ND,^[110–113]^ but the mechanism of interaction with SARS-CoV-2 is poorly documented and needs further study.

GRN by COVID-19 and ND identified miRNAs with key roles, such as hsa-miR-1-3p, hsa-miR-27a-3p, hsa-miR-155-5p, hsa-miR-34a-5p, and hsa-miR-124-3p. These miRNAs were not only expressed in COVID-19 with AD, PD and MS, but also influence the progression of various types of tumor cells. hsa-mir-124-3p accelerates the growth and metastasis of osteosarcoma cells,^[114]^ glioma cells,^[115]^ and hepatocellular carcinoma cells.^[116]^ In addition, the TFs FOXC1, GATA2, JUN, YY1, and RELA were found to be overexpressed in common DEGs of ND and COVID-19 This is consistent with previous findings.^[117–119]^ Unfortunately, the development of JNK subtype inhibitors as treatments for neurodegenerative diseases has not been smooth.

Among the drug prediction results according to Table 5, chloroquinoline was found to be available for ND and COVID-19. In ND, chloroquinoline promoted degradation of Aβ oligomers and inhibited accumulation to restore endocytosis and ameliorate Aβ toxicity and improve AD symptoms^[120]^; improved motor and non-motor deficits in a monkey model of MPTP-induced Parkinson’s disease via the AKT/mTOR pathway^[121]^; and improved behavioral and pathological phenotypes in mice with Huntington’s chorea.^[122]^ In the treatment of COVID-19, Clioquinol and its analogs inhibited the cytopathic effects induced by SARS-CoV-2 infection via in vitro Spike protein interactions.^[123]^ β2 receptor agonist terbutaline is widely used in the treatment of COPD,^[124]^ and recent studies have shown an association between COPD and COVID-19. Contrasting our study, Mahmud SMH’s team considered only 1 GSE dataset for each disease, which is clearly insufficient.^[125]^ Brake^[126]^ demonstrated a link between COPD and increased SARS-CoV-2 viral adhesion and cell entry associated with multiple biomarkers of COPD which may be a risk factor for increased severity of COVID-19, triggering and exacerbating the COVID-19 cytokine storm.^[127]^ This would provide new ideas for clinical prevention and treatment of COVID-19 combined with AD, PD or MS.

**Table 5:** Drug prediction of common DEGs for COVID-19 and ND.

Disease	Drug	Adjusted *P*-value	Combined Score	Molecule	Structure
COVID-19 and AD	Fluorometholone	.01	1525	C_22_ H_29_ FO_4_	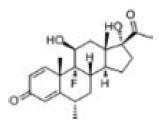
	Terbutaline	.01	1165	C_12_ H_19_ NO_3_	_ 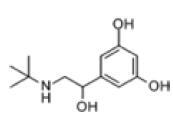 _
	Chinomethionate	.01	1110	C_10_ H_6_ N_2_ OS_2_	_ 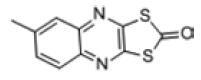 _
	Flumetasone	.01	1110	C_22_ H_28_ F_2_ O_5_	_ 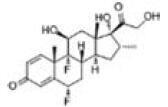 _
	Diflorasone	.01	1060	C_5_ H_9_ Cl_2_ N_3_ O_2_	_ 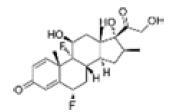 _
COVID-19 and PD	Miglitol	<.001	1262	C_8_ H_17_ NO_5_	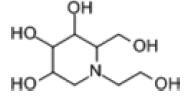
	N-ethylmaleimide	<.001	714	C_6_ H_7_ NO_2_	_ 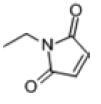 _
	Tarichatoxin	<.001	532	C_17_ H_25_ N_3_ O_15_	_ 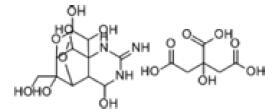 _
	Carisoprodol	<.001	531	C_12_ H_24_ N_2_ O_4_	_ 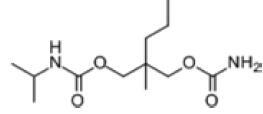 _
	Enkephalin	<.001	455	C_26_ H_33_ I_2_ N_5_ O_6_	_ 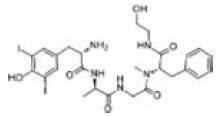 _
COVID-19 and MS	labetalol	<.001	2701	C_19_ H_24_ N_2_ O_3_	_ 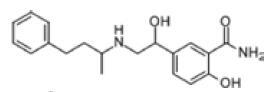 _
	Acetohexamid	<.001	2540	C_15_ H_20_ N_2_ O_4_ S	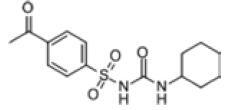
	Clioquinol	<.001	2540	C_9_ H_5_ ClINO	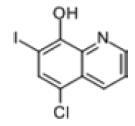
	MIGLITOL	<.001	2299	C_8_ H_17_ NO_5_	_ 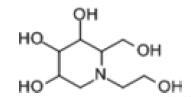 _
	Suloctidil	<.001	2080	C_20_ H_35_ NOS	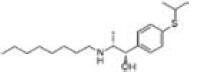

AD = Alzheimer’s disease, COVID-19 = coronavirus disease, DEG = differentially expressed gene, MS= multiple sclerosis, ND = neurodegenerative diseases, PD= Parkinson’s disease.

In the analysis of genetic disease associations, it was shown that there is some association between COVID-19 and ND and psychiatric disorders. Related analyses have examined that there is indeed a correlation between schizophrenia and COVID-19,^[128]^ and that patients with mental health disorders have a higher risk rate of severe COVID-19 infection and mortality after infection compared to patients without mental health disorders.^[129]^ Epidemiological and clinical studies have shown comorbidity between schizophrenia and ND, and Li^[130]^ explored their relationship from a genetic and transcriptomic perspective and found a common genetic background in order to schizophrenia and ND. Associations between other psychiatric disorders and COVID-19 have also been reported.^[131]^

## 5. Conclusion

To explore the pathogenesis and genetic mechanisms of COVID-19 comorbidity with ND, this study identified common DEGs in AD, PD and MS with COVID-19 and its immune response. Integrating common DEGs in ND and COVID-19 and their immune response to identify GO and KEGG pathways, cAMP, adipocytokines, IL-17 MAPK, PI3K-Akt and other immune-related pathways were highly enriched. A PPI network was created to understand the relationship between COVID-19 and ND, and after identifying HGs, the mechanism of action of VIPs therein in COVID-19 and ND was detailed. Transcriptional and post-transcriptional analyses were also performed to reveal the miRNAs and TFs potentially involved and to identify important factors for infection with SARS-CoV-2. Finally, drug prediction was performed to identify potential drug molecules. The genes, pathways and networks identified in this study are consistent with previous studies and suggest multiple common morbid mechanisms between COVID-19 and AD, PD and MS, providing new ideas and therapeutic strategies for the prevention and treatment of COVID-19 and ND in the clinic.

## Acknowledgements

This study was supported by funds from the National Natural Science Foundation of China (No. 81873299, 81973747).

## Author contributions

**Conceptualization:** Fan Bu, Ruiqian Guan.

**Funding acquisition:** Yonghou Zhao, Jianbo Chai.

**Methodology:** Ruiqian Guan.

**Software:** Shijie Yin.

**Visualization:** Zhao Liu.

**Writing – original draft:** Fan Bu, Wanyu Wang.

**Writing** – **review & editing:** Fan Bu.
